# Frameworks for developing and evaluating public health interventions in primary prevention and health promotion: a scoping review

**DOI:** 10.1186/s12889-026-27174-x

**Published:** 2026-04-11

**Authors:** Saskia Muellmann, Tina Jahnel, Núria Pedrós Barnils, Chen-Chia Pan, Ansgar Gerhardus

**Affiliations:** 1 Leibniz Institute for Prevention Research and Epidemiology – BIPS, Bremen, Germany; 2 Leibniz ScienceCampus Digital Public Health (LSC DiPH), Bremen, Germany; 3 Department for Health Services Research, Institute for Public Health und Nursing Sciences – IPP, University of Bremen, Bremen, Germany; 4 Department Prevention and Health Promotion, Institute for Public Health und Nursing Sciences – IPP, University of Bremen, Bremen, Germany; 5 Department of Epidemiology and Global Health, Umeå University, Umeå, Sweden

**Keywords:** Public health, Health promotion, Prevention, Evaluation, Development, Framework, Scoping review

## Abstract

**Background:**

A variety of frameworks for developing and evaluating public health interventions exists. However, an overview of existing public health frameworks is missing posing challenges for practitioners, policymakers, and researchers navigating the dynamic field of public health. This scoping review aims to give an overview of public health frameworks for the development and evaluation of interventions in primary prevention and health promotion.

**Methods:**

This scoping review followed the Joanna Briggs Institute scoping review methodology and adhered to the Preferred Reporting Items for Systematic Reviews and Meta-Analyses Extension for Scoping Reviews (PRISMA-ScR) checklist. Five electronic databases (MEDLINE, Scopus, IEEE Xplore, CINAHL, and PsycINFO; to 12 April 2022) were searched for English-written peer-reviewed journal articles describing descriptive frameworks (i.e., providing standards, principles, criteria, characteristics, or properties) for the development and/or evaluation of interventions focusing on primary prevention and health promotion. In addition, reference lists of included reviews were screened manually. Title/abstract and full-text screening were independently performed by two researchers. Criteria and sub-criteria from the included frameworks were (1) clustered to the HTA Core Model and (2) grouped and summarized into themes and sub-themes using a deductive-inductive iterative approach by one researcher and reviewed by a second researcher.

**Results:**

39 frameworks out of 4,680 records were included in this scoping review. Frameworks were published between 1986 and 2021 with 19 frameworks published in the USA. About half of the frameworks were designed to encompass all public health interventions in primary prevention and health promotion, while the other half were more specific (e.g., disease or behaviour-specific). The number of criteria and sub-criteria in the included frameworks varied widely, ranging from two to eleven criteria and from none to 63 sub-criteria. Criteria and sub-criteria from the 39 included frameworks were grouped into seven themes: (1) intervention development, (2) intervention implementation, (3) intervention evaluation, (4) dissemination and sustainability, (5) organizational aspects, (6) ethics, and (7) context.

**Conclusions:**

This scoping review provides an overview of existing public health frameworks and identified seven themes, organized into four phases and three non-phase-related themes, for developing and evaluating public health interventions in prevention and health promotion.

**Supplementary Information:**

The online version contains supplementary material available at 10.1186/s12889-026-27174-x.

## Background

Public health interventions play a crucial role in addressing emerging health challenges and promoting the well-being of populations. As the field of public health continues to adapt to dynamic health threats and societal changes, there is a growing need for robust frameworks to guide the systematic development, implementation, and evaluation of interventions [1]. These frameworks are essential for guiding the systematic planning, implementation, and assessment of interventions to improve health outcomes and reduce community disparities [2].

Existing public health frameworks are typically designed with a specific focus on key aspects such as development and evaluation within the field. Development frameworks emphasize creating and enhancing public health strategies, policies, and infrastructure, thus facilitating continuous improvement [3]. Evaluation frameworks, on the other hand, offer systematic approaches to assess the impact and effectiveness of public health programs, helping stakeholders understand the outcomes and make informed decisions [4]. Additionally, there are frameworks tailored to specific public health interventions, concentrating on areas such as disease prevention [5], health promotion [6, 7], crisis response [8, 9], or infodemic management [10]. These specialized frameworks recognize the diverse nature of public health challenges and enable a more nuanced and targeted approach to address them.

Whilst there are various frameworks for developing and evaluating public health interventions [1, 11–14], an overview of existing public health frameworks to date is missing. A review of academic and grey literature published in 2017 included 98 documents of evaluation guidance for public health interventions [15]. However, these did not relate to the development of public health interventions, and the information included in the documents was not synthesized. In addition, reviews have been published summarising specific frameworks for implementation [16] and optimization of public health interventions [17], translating research evidence into practice [18], or applying evaluation frameworks for specific public health programs (e.g., physical activity) [19]. The absence of a consolidated overview poses a challenge for practitioners, policymakers, and researchers navigating the dynamic field of public health.

This scoping review aims to give an overview of the currently existing landscape of public health frameworks for developing and evaluating interventions in primary prevention and health promotion. It should support policymakers, researchers, and practitioners in identifying a framework that best fits their needs and interests. We believe that this comprehensive understanding will not only aid in navigating the existing diversity of frameworks but may also contribute to developing more effective, adaptable, and contextually relevant strategies in public health interventions.

## Methods

### Study design and protocol

This scoping review followed the Joanna Briggs Institute (JBI) scoping review methodology [20] and adhered to the Preferred Reporting Items for Systematic Reviews and Meta-Analyses Extension for Scoping Reviews (PRISMA-ScR) checklist [21]. The PRISMA-ScR checklist is available in Supplementary Material 1. A protocol for a scoping review on public health and digital health frameworks was prospectively registered [22]. In this paper, we focus on the results of the public health frameworks.

### Eligibility criteria

The eligibility criteria for this scoping review are listed in Table 1 and were derived from the Population, Concept, and Context (PCC) criteria. This scoping review included English-written academic literature describing frameworks to support the systematic development or evaluation of health interventions (Concept) focusing on primary prevention and health promotion (Context) at a population level (Population). Eligible public health frameworks are needed to describe properties, characteristics, and/or qualities of the development or evaluation of health interventions (i.e., descriptive frameworks [23]). Frameworks were chosen over theories, guidelines or models since we understand frameworks in health sciences as comprehensive tools that allow for the interaction of multiple levels of structures and are adaptable to various contexts [24, 25]. On the other hand, we understand models as tools that focus on specific relationships and processes tailored to certain scenarios, theories as tools that aim to explain a phenomenon’s underlying factors (like health outcomes) [26] and guidelines as sequential steps (i.e., not descriptive but instructive). In addition, empirical applications (e.g., applying a framework for evaluating a public health intervention or program) of frameworks were excluded if they did not describe a new framework or make changes to an existing framework. Eligible publication types included peer-reviewed journal articles of any study design whereas comments, corrections, letters, editorials, oral presentations, and posters were excluded because they do not generally describe a newly developed or modified framework.

**Table 1:** Eligibility criteria

Inclusion criteria	Exclusion criteria
Population	
• General population.	• Populations in diagnostic, therapeutic, curative, pharmaceutical, surgical, clinical, or rehabilitation settings.
Concept	
• A descriptive framework, i.e., a framework that outlines specific standards, principles, criteria, characteristics, or properties to determine the quality or integrity of health interventions [23].	• No framework reported • Framework does not provide specific standards, principles, criteria, characteristics, or properties. • Empirical applications of frameworks if they did not describe a new framework or make changes to an existing framework
• Framework aims to develop, monitor, validate, evaluate, or implement interventions related to public health. An intervention can be a policy, program, project, or activity.	• Framework does not focus on developing, monitoring, validating, evaluating, or implementing health interventions. • No intervention described.
Context	
• The intervention is related to public health with a focus on primary prevention or health promotion.	• The intervention is not related to public health with a focus on primary prevention or health promotion, e.g., diagnostic, therapeutic, curative, pharmaceutical, surgical, clinical, rehabilitation interventions.
Publication type and language	
• Publication type: peer-reviewed journal articles of any study design	• Other publication types: comment, correction, letter, editorial, protocol, oral presentation, or poster
• Language: English	• Other languages than English

### Search strategy and databases

Journal articles were identified using an electronic literature search of international bibliographic databases and a manual search of reference lists of included systematic and scoping reviews. The following bibliographic databases were searched from inception to 12 April 2022, with no language or publication date limitations:

1. MEDLINE (via PubMed)
2. Scopus
3. IEEE Xplore
4. CINAHL (via EBSCO)
5. PsycINFO (via Ovid)

### Search

The initial search syntax in the bibliographic databases was structured around three primary keywords: “Public Health” [Title/Abstract] AND Evaluation [Title] AND Framework [Title], combined using Boolean operators During the search development process, test searches indicated that including “evaluation” and “framework” in the abstract field resulted in a high number of irrelevant records, as these terms were frequently mentioned in the background or methods sections without indicating that the study itself described an evaluation framework. To improve precision, we restricted these terms (and their synonyms) to the title field while maintaining “public health”, “health promotion”, and “prevention” in both the title and abstract fields to enhance sensitivity for identifying relevant subject areas. MeSH terms were not used for the consideration that “public health” is overly broad, “evaluation” lacks specificity for identifying relevant frameworks, and no precise equivalent exists for “framework” in the MeSH hierarchy. Instead, a targeted keyword-based approach was employed, with synonyms, truncation, and wildcard characters used where appropriate to balance sensitivity and specificity. To further ensure comprehensiveness, we conducted manual reference screening of relevant reviews to identify evaluation frameworks that may not have been captured by the search strategy, including studies using alternative terminology such as “health education”. The final search syntax was adapted for each bibliographic database, incorporating database-specific search fields and subject terms. The strategy was reviewed and approved by a professional librarian (Lara Christianson, refer to Acknowledgments). One researcher (C-CP) conducted the electronic search in bibliographic databases with no language or publication date limitations. The search syntax for each database can be found in Supplementary Material 2.

### Selection of sources of evidence

The electronic literature search results were uploaded to the online systematic review management software Covidence (Melbourne, Australia: Covidence). After deduplication, titles and abstracts of the records were independently assessed by two researchers for eligibility (SM, NPD, C-CP, Dorothee Jürgens, Sarah Janetzki, Sarah Forberger, and Jonathan Kolschen; refer to Acknowledgments). To ensure that inclusion and exclusion criteria were applied correctly, the first step, titles and abstracts were assessed jointly by all researchers involved in the screening process. In addition, regular meetings were held to discuss the screening progress. In the second step, full-texts of potentially relevant records were obtained and reviewed independently by two researchers (SM, NPD, C-CP, Dorothee Jürgens, Sarah Janetzki, Sarah Forberger, and Jonathan Kolschen; refer to Acknowledgments). Any disagreements between the two researchers in titles and abstracts and full-text screening stages were resolved by discussion until a consensus was reached. If no consensus was reached, a third researcher was involved to make the final decision (SM, NPD, or C-CP). A manual search of the reference lists of the included systematic or scoping reviews was conducted by one researcher (C-CP) to identify further relevant frameworks. All researchers involved in the screening process were experienced in conducting systematic reviews and held a PhD or Master’s degree, except for one student assistant.

### Data charting process and items

A data coding sheet was developed a-priori in Microsoft Excel. General information was extracted from the included public health frameworks, including bibliographic information (i.e., first author, study title, year of publication, and country of corresponding author), methods applied in developing the framework (i.e., framework’s development method), and framework characteristics (i.e., name, purpose, and function of framework, and number of criteria and sub-criteria). Data coding was performed by one researcher (SM, NPD, C-CP, Dorothee Jürgens, Sarah Janetzki, Sarah Forberger, or Jonathan Kolschen; refer to Acknowledgments). For quality assurance, a random sample of ten included frameworks was coded by two researchers (SM, NPD, or C-CP) as pilot testing for the coding process. The data coding sheet was revised in an iterative process during the pilot coding and calibrated within the team.

### Quality assessment

According to the PRISMA-ScR checklist [21], the JBI scoping review methodology [20], and the framework proposed by Arksey and O’Malley [27], a quality assessment of included articles was not performed.

### Data synthesis

Firstly, all criteria and sub-criteria from the included frameworks were deductively clustered into the domains suggested by the EUnetHTA Technology Assessment (HTA) Core Model [28] using Microsoft Excel. The HTA Core Model consists of nine domains (i.e., health problem and current use of technology, description and technical characteristics of technology, safety, clinical effectiveness, costs and economics, ethics, organizational aspects, patients and social aspects, and legal aspects) with over 50 sub-domains [28] and was used as a first orientation to systematically structure the criteria and sub-criteria from the included frameworks. We chose the HTA Core Model as a starting point because it has been developed through a collaboration of multiple organizations and institutions over more than ten years, during which it was field-tested and refined in many iterations [29]. Framework criteria and sub-criteria that did not fit any of the HTA Core Model domains and sub-domains were inductively clustered into new domains and sub-domains. HTA domains and sub-domains that were irrelevant for the purpose of this subject were deleted. Secondly, framework criteria and sub-criteria were grouped and summarized into themes and sub-themes. All steps (i.e., clustering, grouping, and summarizing) were performed by one researcher (SM or NPD) and reviewed by a second researcher (SM or NPD) using a deductive-inductive iterative approach with several feedback loops between the two involved researchers. Any disagreements between the two researchers were resolved by discussion until a consensus was reached. If no consensus was reached, a third researcher (C-CP) was involved to make the final decision.

## Results

### Study selection

**Fig. 1:**
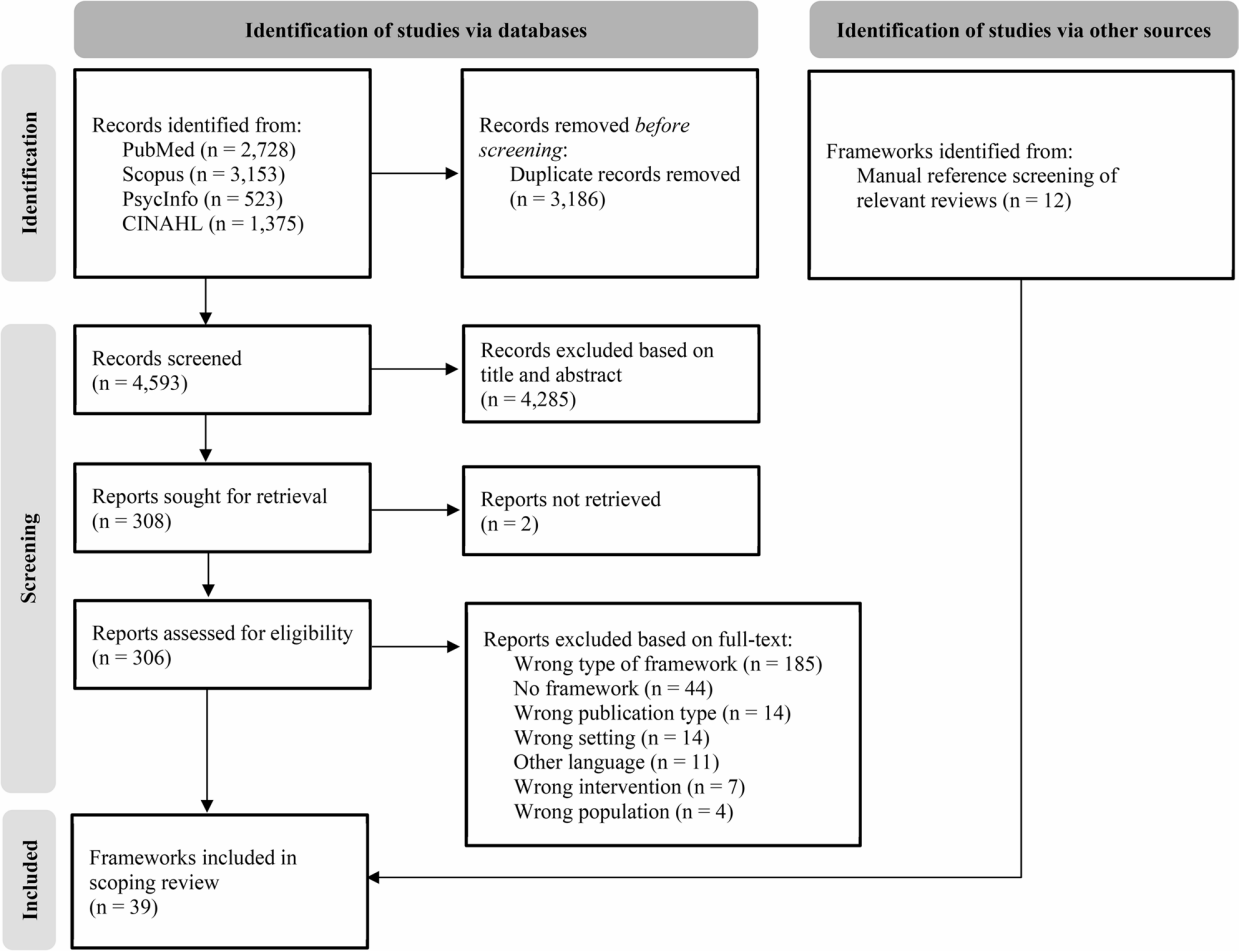
PRISMA Flowchart

This review includes data from 39 frameworks from original research articles that met the eligibility criteria out of 4,593 records identified in the electronic literature search after deduplication and 87 additional records identified from screening references list of included reviews (Fig. 1).

### Characteristics of included frameworks

The 39 frameworks included in the scoping review were published between 1986 and 2021 (Table 2). One framework was published in the 1980s [30], two frameworks were published in the 1990s [12, 31], eight frameworks were published in the 2000s [32–39], 25 frameworks were published in the 2010s [11, 40–63], and three from 2020 onwards [64–66]. Almost half of the frameworks (*n* = 19) were published in the USA [11, 12, 30–32, 34, 35, 38, 40, 42, 46, 48, 49, 51, 53, 54, 56, 58, 60], ten were published in European countries [37, 43, 55, 57, 59, 61–65], three were published in Australia [33, 44, 50], two each were published in Canada [36, 39] and India [47, 66], and one each in Iran [41], Mexico [52], and a consortium of seven countries from Latin America [45].

**Table 2:** Study characteristics of included frameworks

Study (Author, year)	Country	Name of the framework	Purpose of the framework	Framework’s development method^1^, developers^2^, function^3^	N° criteria (sub-criteria)
Aguirre 2013 [40]	USA	Development Guidelines from a Study of Suicide Prevention Mobile Applications (Apps)	To develop, implement or evaluate mobile applications for suicide prevention for underserved populations	S, R, D, I, E	4 (12)
Akrami 2018 [41]	Iran	An ethical framework for evaluation of public health plans: a systematic process for legitimate and fair decision-making	To guide the ethical evaluation of public health programs and interventions	S, L, R, OP, E	5 (18)
Baranowski 2000 [32]	USA	Process Evaluations of the 5-a-Day Projects	To evaluate components of process evaluation in interventions that aim for behaviour change	N, NA, I, E	11 (39)
Bryant 2014 [42]	USA	Community-Based Prevention Marketing for Policy Development: A New Planning Framework for Coalitions	To assist the needs of diverse coalitions addressing public health issues	N, NA, E	5 (20)
Bunde-Birouste 2007 [33]	Australia	Strengthening peace-building through health promotion development of a framework	To provide a rapid assessment of the peace-building/conflict prevention components of health initiatives in precarious, unstable settings	S, R, D, V	5 (29)
Cavill 2012 [43]	UK	Standard Evaluation Framework for physical activity interventions	To describe and explain the information that should be collected in any evaluation of an intervention that aims to increase participation in physical activity	N, NA, E	7 (52)
Centers for Disease Control and Prevention 1999 [12]	USA	Framework for program evaluation in public health	To systematically organize the essential elements of a program to improve and account for public health actions	S, O, R, OP, E	4 (30)
Chinman 2001 [34]	USA	Using the Getting to Outcomes (GTO) Model in a Statewide Prevention Initiative	To help practitioners formulate high-quality planning, implementation, and evaluation strategies for programs and policies	N, R, D, M, E	10 (0)
Cunningham 2019 [44]	Australia	Tackling the wicked problem of health networks: the design of an evaluation framework	To evaluate the effectiveness and sustainability of clinical and health networks	L, R, OP, E	3 (20)
Eslava-Schmalbach 2019 [45]	Latin American countries	Conceptual framework of equity-focused implementation research for health programs (EquIR)	To implement programs, policies or health interventions with a focus on equity	L, R, OP, M, V, E	4 (18)
Glasgow 1999 [31]	USA	Evaluating the public health impact of health promotion interventions: The RE-AIM framework	To evaluate the public health impact of health promotion interventions	N, L, R, M, E	5 (0)
Glasgow 2019 [11]	USA	RE-AIM planning and evaluation framework: Adapting to new science and practice with a 20-year review	To plan, evaluate, and implement public health interventions	N, L, R, D, M, E	8 (11)
Goldsmith 2013 [46]	USA	Thinking Inside the Box: The Health Cube Paradigm for Health and Wellness Program Evaluation and Design	To design, evaluate, and improve initiatives to provide optimal value in each step of a health promotion program	N, NA, D, E	3 (15)
Gopichandran 2012 [47]	India	Monitoring 'monitoring' and evaluating 'evaluation': an ethical framework for monitoring and evaluation in public health	To build the capacity of ethics committees to review monitoring and evaluation proposals	O, R, V, E	5 (18)
Green 1986 [30]	USA	Evaluation Model: A Framework for the Design of Rigorous Evaluation of Efforts in Health Promotion	To identify the objects of interest, classify the comparison methods under standards of acceptability or rules of evidence	N, NA, E	6 (18)
Hoddinott 2010 [48]	USA	Group interventions to improve health outcomes: a framework for their design and delivery	To encompass a systematic approach to designing, evaluating, and reporting interventions in group settings	S, L, R, D, I, E	3 (15)
Huff 2015 [49]	USA	The Cultural Assessment Framework	To enhance a better understanding of the similarities and differences between the mainstream culture and the specific cultural or ethnic group targeted for intervention	N, R, D	5 (25)
Kegler 2000 [35]	USA	Assessing Community Change at Multiple Levels: The Genesis of an Evaluation Framework for the California Healthy Cities Project	To synthesize current thinking and practice on the evaluation of community projects	S, R, OP, E	5 (28)
Klepac Pogrmilovic 2019 [50]	Australia	The development of the Comprehensive Analysis of Policy on Physical Activity (CAPPA) framework	To improve the comprehensiveness and contribute to the standardization of physical activity policy analysis research	S, L, O, R, OP, D, E	6 (38)
Leeman 2012 [51]	USA	An Evaluation Framework for Obesity Prevention Policy Interventions	To evaluate public policy at different levels	O, R, E	4 (15)
León-Castañeda 2019 [52]	Mexico	Electronic health (e-Health): a conceptual framework for its implementation in health services facilities	To analyse the implementation of e-Health components and their effects on the quality of health services provision	N, R, M, E	6 (29)
McLees 2015 [53]	USA	Defining and assessing quality improvement outcomes: A framework for public health	To identify types of improvement outcomes targeted by public health quality improvement efforts and their impact on public health practice	L, R, E	2 (13)
Noar 2012 [54]	USA	An Audience–Channel–Message–Evaluation (ACME) Framework for Health Communication Campaigns	To ensure careful thought at each step in a public health campaign design process	N, R, D, M, E	4 (22)
Pfadenhauer 2017 [55]	Germany	Making sense of complexity in context and implementation: the Context and Implementation of Complex Interventions (CICI) framework	To develop a framework to facilitate the structured and comprehensive conceptualisation and assessment of context and implementation of complex interventions	L, O, R, M	4 (23)
Poland 2009 [36]	Canada	Settings for health promotion: An analytic framework to guide intervention design and implementation	To systematically analyse features of settings that can have the strongest impact on intervention design and delivery	O, R, M, E	3 (11)
Proctor 2011 [56]	USA	Outcomes for Implementation Research: Conceptual Distinctions, Measurement Challenges, and Research Agenda	To advance the vocabulary of implementation science around implementation outcomes	S, L, R, M, E	8 (0)
Roberts 2009 [37]	UK	Standard Evaluation Framework for Weight Management Interventions	To describe and explain the information that should be collected in any evaluation of a weight management intervention	N, NA, E	5 (63)
Roberts 2012 [57]	UK	Standard Evaluation Framework for dietary interventions	To describe and explain the information that should be collected in any evaluation of an intervention that aims to improve dietary intake or associated behaviour	N, NA, E	7 (52)
Saunders 2005 [38]	USA	Developing a Process-Evaluation Plan for Assessing Health Promotion Program Implementation: A How-To Guide	To develop a process-evaluation plan for any health-promotion program	N, R, E	7 (0)
Shekelle 2013 [58]	USA	AHRQ Methods for Effective Health Care	To inform efficacious, effective, sustainable global health programs at the community and scale level	S, L, R, OP, D	5 (10)
Stratil 2020 [64]	Germany	Development of the WHO‑INTEGRATE evidence‑to‑decision framework: an overview of systematic reviews of decision criteria for health decision‑making	To provide a comprehensive overview of criteria used in or proposed for real-world decision-making processes	L, R, D	7 (42)
Thurston 2003 [39]	Canada	Development and testing of a framework for assessing the effectiveness of health promotion	To assess the overall effectiveness of health promotion in one Canadian province	O, R, E	9 (42)
Van Weert 2011 [59]	Europe and Africa	Dance4life: Evaluating a global HIV and AIDS prevention program for young people using the Pre-Im framework for process evaluation	To evaluate the process and effectiveness of HIV and AIDS prevention programs	O, R, E	6 (13)
Vanderkruik 2017 [60]	USA	A Contextual Factors Framework to Inform Implementation and Evaluation of Public Health Initiatives	To evaluate contextual factors affecting an initiative at multiple phases of its life cycle,including design, implementation, scale-up, spread, and sustainability	O, OP, M, E	4 (11)
Wagemakers 2010 [61]	The Netherlands	Coordinated action checklist: a tool for partnerships to facilitate and evaluate community health promotion	To facilitate and evaluate coordinated action in community health promotion	O, R, OP, D, E	6 (25)
WHO 2010 [62]	Switzerland	Nine steps for developing a scaling-up strategy	To outline a concise, step-by-step process for developing a scaling-up strategy	N, OP, D, M, V, E	9 (20)
Wienert 2021 [65]	Germany	Implementing Health Apps for Digital Public Health – An Implementation Science Approach Adopting the Consolidated Framework for Implementation Research	To help develop successful implementation plans and models for health apps and show the complexity of a successful implementation	O, R, M	5 (25)
Wolfenstetter 2011 [63]	Germany	Conceptual Framework for Standard Economic Evaluation of Physical Activity Programs in Primary Prevention	To manage, conduct and implement an economic evaluation embedded in standardized methods for physical activity programs in primary prevention	L, R, I, M	8 (23)
Zadey 2021 [66]	India	Ethics-driven policy framework for implementation of movement restrictions in pandemics	To bring an ethically sound approach to movement-restrictive public health interventions in the context of covid-19	N, R, V, E	11 (34)

^1^(N) non-empirical, (S) structured-survey/interview method, (L) literature review, (O) other methods, ^2^(R) researchers, (OP) other professionals, (NS) not stated, ^3^(D) development, (I) implementation, (M) monitoring, (V) validation (E) evaluation

Five of the included frameworks were developed based on a literature review [44, 45, 53, 63, 64] and three were developed using a structured survey/interview [33, 35, 40]. Eight frameworks used other methods such as knowledge gathered from a previous project [36], case studies (e.g., consensus by experts and tested on ongoing projects) [39, 47], applying a realist evaluation approach [60], applying an action research approach (i.e., participatory research approach with experts) [61], adapting an existing framework (i.e., the Consolidated Framework for Implementation Research [67]) [65], or by combining two (i.e., the Reach, Effectiveness, Adoption, Implementation, and Maintenance (RE-AIM) framework [31] and the Implementation of Change in Health Care model [68]) [59] or more existing frameworks (i.e., theories and frameworks related to policy making and evaluation in public health) [51]. Nine frameworks employed more than one method for developing the framework (e.g., any combination of literature review, structured interview/survey, or other method) [11, 12, 31, 41, 48, 50, 55, 56, 58]. For instance, Klepac Pogrmilovic and colleagues [50] used a structured survey/interview, literature review, and other methods to develop their framework. 14 frameworks described no process for the framework development [30, 32, 34, 37, 38, 42, 43, 46, 49, 52, 54, 57, 62, 66]. 22 frameworks were developed by researchers [11, 31, 33, 34, 36, 38–40, 47–49, 51–56, 59, 63–66] and two frameworks by other professionals (e.g., health organization) [60, 62]. Eight frameworks were developed by researchers and other professionals including experts (e.g., ethics, health equity, public health, evaluation, methods) [12, 41, 45, 58], public health program directors, managers and staff [12], health officials and institutions [12, 44, 61], (policy) decision makers [45, 50, 58], and city representatives and partnerships [35, 61]. In seven frameworks the developer was not stated [30, 32, 37, 42, 43, 46, 57].

Most of the included frameworks aimed at evaluating interventions (*n* = 32) [11, 12, 30–32, 34–48, 50–54, 56, 57, 59–62, 66], followed by developing (*n* = 13) [11, 33, 34, 40, 46, 48–50, 54, 58, 61, 62, 64], and monitoring interventions (*n* = 13) [11, 31, 34, 36, 45, 52, 54–56, 60, 62, 63, 65]. Fewer frameworks aimed at validating (i.e., ensuring feasibility of intervention prior to or during early implementation) (*n* = 5) [33, 45, 47, 62, 66] or implementing interventions (*n* = 4) [32, 40, 48, 63]. About half of the frameworks (*n* = 20) were designed for more than one function (e.g., development, implementation, monitoring, validation, or evaluation) [11, 31–34, 36, 40, 45–48, 50, 52, 54, 56, 60–63, 66]. To illustrate, Zadey’s framework [66] aims at validating and evaluating movement-restrictive public health interventions in the context of COVID-19 from an ethically point of view.

About half of the frameworks (*n* = 20) addressed generic (non-specific) public health interventions in primary prevention and health promotion [11, 12, 31, 34, 35, 40, 42, 44, 48, 51–56, 58, 60, 62, 64, 65]. For example, Glasgow and colleagues [31] developed the RE-AIM (Reach, Efficacy, Adoption, Implementation, Maintenance) framework that was intended at evaluating the public health impact of health promotion interventions. Fourteen frameworks aimed at specific diseases or behaviors [30, 32, 33, 36–39, 43, 46, 50, 57, 59, 61, 63]. For instance, Cavill and colleagues [43] provide guidance on the information needed to evaluate an intervention targeting physical activity participation. Furthermore, five frameworks were centred on equity and diversity aspects [41, 45, 47, 49, 66] such as Eslava-Schmalbach and colleagues [45] who present a conceptual framework for implementing equity-focused programs, policies, or health interventions.

The number of criteria reported in the included frameworks ranged between two [53] and eleven [32, 66]. Five frameworks applied between nine and eleven criteria [32, 34, 39, 62, 66], seven frameworks seven or eight criteria [11, 38, 43, 56, 57, 63, 64], 15 frameworks five or six criteria [30, 31, 33, 35, 37, 41, 42, 47, 49, 50, 52, 58, 59, 61, 65], and 12 frameworks between two to four criteria [12, 36, 40, 44–46, 48, 51, 53–55, 60]. The number of sub-criteria ranged between none [31, 34, 38, 56] and 63 [37]. Three frameworks had 46 to 63 sub-criteria [37, 43, 57], five frameworks had 31 to 45 sub-criteria [32, 39, 50, 64, 66], 17 frameworks had 16 to 30 sub-criteria [12, 30, 33, 35, 41, 42, 44, 45, 47, 49, 52, 54, 55, 61–63, 65], and 14 frameworks had none to 15 sub-criteria [11, 31, 34, 36, 38, 40, 46, 48, 51, 53, 56, 58–60].

### Themes and sub-themes identified from included frameworks

**Fig. 2:**
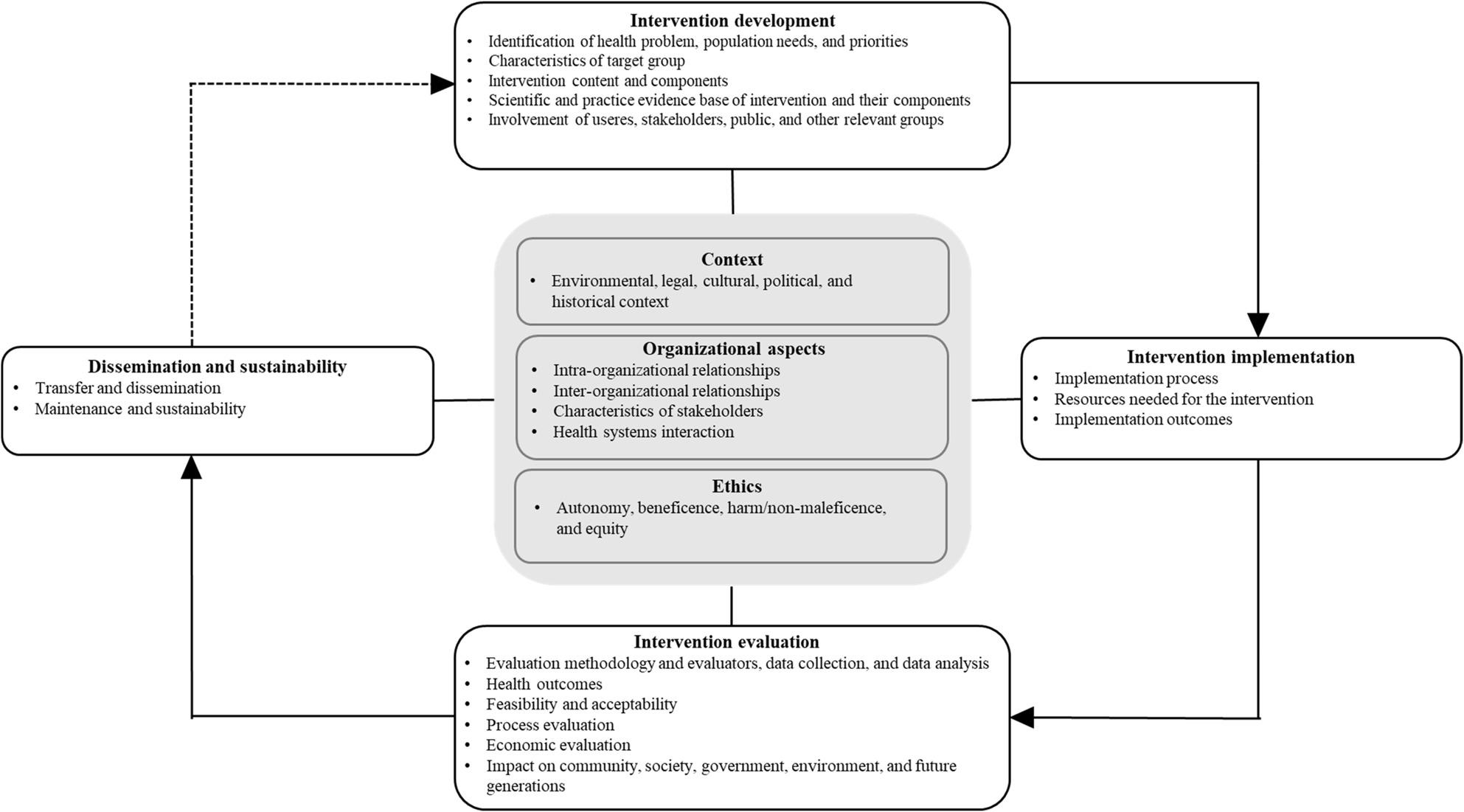
Visual representation of themes and sub-themes identified from included frameworks

Criteria and sub-criteria from the 39 included frameworks were grouped into seven overarching themes: (1) intervention development, (2) intervention implementation, (3) intervention evaluation, (4) dissemination and sustainability, (5) organizational aspects, (6) ethics, and (7) context (Table 3). A visual representation of the themes identified from the 39 included frameworks can be found in Fig. 2, and a detailed description of all extracted (sub)criteria is available in Supplementary Material 3.

**Table 3:** Themes and sub-themes identified from included frameworks (*n* = 39)

Theme	Sub-theme	*N*° of frameworks referring to theme/sub-theme [references]
Intervention development		24 [30, 32, 34–37, 39, 40, 43–46, 48–51, 55, 57, 58, 62–66]
	Identification of health problem, population needs, and priorities	13 [34–37, 39, 43, 45, 46, 50, 51, 57, 58, 64]
	Characteristics of the target group (e.g., sociodemographic characteristics such as age, gender, ethnicity, occupation, living conditions; distribution of diseases/conditions in the target group; values, motivation, engagement, specific competencies of the target group)	12 [34–37, 43, 48, 49, 55, 57, 58, 64, 65]
	Intervention content and components	15 [30, 32, 34, 37, 39, 43, 44, 48, 50, 55, 57, 62–65]
	Scientific and practice evidence base of intervention and their components	13 [32, 34, 36, 37, 39, 40, 43, 48, 55, 57, 58, 65, 66]
	Involvement of users, stakeholders, public, and other relevant groups	12 [35, 36, 39, 41, 45, 46, 48, 50, 58, 61, 62, 65]
Intervention implementation		27 [11, 31, 32, 34, 36–38, 43, 45, 46, 48–59, 62–66]
	Implementation process (e.g., intervention delivery, implementation stages and strategies, implementation difficulties, implementation theory)	9 [11, 32, 45, 52, 54, 58, 64–66]
	Resources needed for implementing the intervention (e.g., human resources such as personnel, infrastructure and equipment such software/hardware, rooms or materials, financial resources)	17 [32, 34, 36, 37, 43, 45, 46, 48, 49, 55–58, 62–65]
	Implementation outcomes (e.g., adoption, reach, and fidelity)	15 [11, 31, 32, 34, 38, 45, 46, 51–53, 55, 56, 59, 63, 65]
	Implementation (without further specification)	1 [50]
Intervention evaluation		32 [11, 12, 30–34, 36, 37, 39–53, 56–58, 62–66]
	Evaluation methodology/strategy and evaluators, data collection, and data analysis	12 [12, 34, 36, 37, 39, 43, 45, 47, 50, 57, 62, 63]
	Health outcomes (e.g., decreased prevalence or incidence of disease, reduction of mortality, change in health behavior, well-being)	15 [11, 30, 31, 33, 36, 37, 39, 43, 44, 46, 48, 49, 53, 57, 63, 66]
	Feasibility	6 [12, 32, 45, 52, 56, 65]
	Process evaluation	9 [33, 37, 40, 43, 46, 50, 53, 57, 62]
	Acceptability (i.e., by users, those delivering the intervention, and society)	13 [12, 37, 40, 43, 45, 47, 49, 50, 53, 56, 57, 64, 65]
	Economic evaluation (e.g., costs, cost-effectiveness, cost-utility, cost-minimization)	14 [11, 12, 36, 37, 43, 44, 51, 53, 57, 58, 63–66]
	Impact of the intervention on the community, society, government, environment, future generations	9 [12, 33, 39, 41, 42, 44, 46, 63, 64]
Dissemination and sustainability		23 [11, 12, 32–34, 36, 37, 39, 41, 43–48, 50–53, 56, 57, 61, 62]
	Transfer and dissemination (i.e., how to communicate intervention outcomes/results and learnings to the target group, public, stakeholders, and government)	11 [12, 36, 39, 41, 43, 44, 47, 50, 53, 57, 62]
	Maintenance and sustainability (e.g., regards of intervention’s maintenance)	18 [11, 32–34, 37, 39, 41, 43, 45, 46, 48, 50–52, 56, 57, 61, 62]
Organizational aspects		18 [11, 12, 35, 36, 39, 41, 42, 44, 47, 49, 50, 52, 59–62, 64, 65]
	Intra-organizational relationships (e.g., roles among stakeholders, communication structures, common goals)	11 [11, 12, 35, 39, 42, 44, 47, 49, 59–61]
	Inter-organizational relationships (e.g., collaboration with external stakeholders at all intervention levels)	12 [35, 36, 39, 41, 42, 44, 50, 60–62, 64, 65]
	Characteristics of stakeholders (i.e., description of features needed for intervention stakeholders)	5 [36, 50, 52, 61, 64]
	Health systems interaction (i.e., how does the intervention interact with the health system)	4 [49, 52, 62, 64]
Ethics		18 [11, 12, 31, 33, 36, 37, 39, 41, 43–46, 52, 55–57, 64, 66]
	Autonomy (i.e., user’s right to self-determination and decision-making)	6 [37, 41, 43, 44, 57, 66]
	Beneficence (i.e., the intervention promotes the good and well-being of intervention users)	7 [11, 12, 31, 36, 41, 46, 64]
	Harm/non-maleficence (i.e., the intervention will cause the least amount of harm to attain beneficial results)	5 [12, 41, 52, 64, 66]
	Equity (i.e., the intervention promotes the fair distribution of resources and opportunities)	9 [12, 33, 39, 41, 43, 45, 52, 57, 64]
	Ethics (without further specifications)	2 [55, 56]
Context		25 [11, 12, 30, 32, 33, 35–41, 43, 47–50, 55, 57, 58, 60, 62, 64–66]
	Environmental context (e.g., from close physical surroundings to bigger geographical resources)	11 [30, 35, 36, 39, 43, 48, 49, 55, 57, 60, 62]
	Legal context including data protection/data security	8 [40, 41, 47, 48, 55, 64–66]
	Cultural context	4 [33, 35, 49, 55]
	Political context	11 [11, 12, 33, 35–37, 43, 50, 55, 57, 58]
	Historical context	1 [36]
	General context (i.e., not specified context)	2 [32, 38]

### Intervention development

The theme *Intervention development* describes the whole process of designing and planning an intervention. 24 of the 39 (62%) included frameworks described criteria for the theme intervention development [30, 32, 34–37, 39, 40, 43–46, 48–51, 55, 57, 58, 62–66]. The theme comprised five sub-themes referring to the identification of the health problem, population needs, and priorities (*n* = 13) [34–37, 39, 43, 45, 46, 50, 51, 57, 58, 64], the description of characteristics of the target group (*n* = 12) [34–37, 43, 48, 49, 55, 57, 58, 64, 65], the intervention content and components (*n* = 15) [30, 32, 34, 37, 39, 43, 44, 48, 50, 55, 57, 62–65], the scientific evidence base of the intervention (*n* = 13) [32, 34, 36, 37, 39, 40, 43, 48, 55, 57, 58, 65, 66], and the involvement of users, stakeholders, and public in the intervention development process (*n* = 12) [35, 36, 39, 41, 45, 46, 48, 50, 58, 61, 62, 65].

### Intervention implementation

The theme *Intervention implementation* includes aspects to consider before and during implementing the intervention into the health care system. Criteria for implementing interventions were described in 27 of the 39 (69%) included frameworks [11, 31, 32, 34, 36–38, 43, 45, 46, 48–59, 62–66]. Nine frameworks described criteria for the implementation process, such as intervention delivery, implementation stages and strategies, implementation difficulties, or implementation theory [11, 32, 45, 52, 54, 58, 64–66]. Seventeen frameworks included criteria for the resources needed for implementing the intervention, such as human resources or infrastructure and equipment [32, 34, 36, 37, 43, 45, 46, 48, 49, 55–58, 62–65]. Implementation outcomes such as adoption, reach, and fidelity were described in 15 frameworks [11, 31, 32, 34, 38, 45, 46, 51–53, 55, 56, 59, 63, 65]. In one framework implementation was mentioned without further specification [50].

### Intervention evaluation

The theme *Intervention evaluation* describes which evaluation methods were used and whether the intervention works as intended. 82% (*n* = 32) of the included frameworks described criteria for intervention evaluation [11, 12, 30–34, 36, 37, 39–53, 56–58, 62–66]. Intervention evaluation was divided into seven sub-themes. Twelve frameworks described criteria for evaluation methods, data collection, and data analysis [12, 34, 36, 37, 39, 43, 45, 47, 50, 57, 62, 63]. Feasibility was described in six frameworks [12, 32, 45, 52, 56, 65] and process evaluation in nine frameworks [33, 37, 40, 43, 46, 50, 53, 57, 62]. Health outcomes such as decreased prevalence or incidence, or change in health behavior were mentioned in 15 frameworks [11, 30, 31, 33, 36, 37, 39, 43, 44, 46, 48, 49, 53, 57, 63, 66]. Criteria referring to the acceptability of the intervention by users, those delivering the intervention, or society were described in 13 frameworks [12, 37, 40, 43, 45, 47, 49, 50, 53, 56, 57, 64, 65]. Economic evaluation was addressed in 14 frameworks [11, 12, 36, 37, 43, 44, 51, 53, 57, 58, 63–66]. The impact of the intervention on the community, society, government, environment, or future generations was considered in nine frameworks [12, 33, 39, 41, 42, 44, 46, 63, 64].

### Dissemination and sustainability

*Dissemination and sustainability* describe how intervention results were communicated and how the intervention is transferred and maintained in real-world settings. The theme of dissemination and sustainability comprised the sub-themes transfer and dissemination and maintenance and sustainability and was reported in 23 of the 39 (59%) included frameworks [11, 12, 32–34, 36, 37, 39, 41, 43–48, 50–53, 56, 57, 61, 62]. Transfer and dissemination include the communication of intervention outcomes and results and learnings to the target group, public, stakeholders, and government (*n* = 11) [12, 36, 39, 41, 43, 44, 47, 50, 53, 57, 62]. Criteria for intervention maintenance were reported in 18 frameworks [11, 32–34, 37, 39, 41, 43, 45, 46, 48, 50–52, 56, 57, 61, 62].

### Organizational aspects

The theme *Organizational aspects* includes criteria and sub-criteria from the included frameworks that consider structural aspects of the context wherein an intervention is developed and implemented, alongside the role of the various stakeholders involved. 18 of the 39 (46%) included frameworks included criteria that addressed organizational aspects [11, 12, 35, 36, 39, 41, 42, 44, 47, 49, 50, 52, 59–62, 64, 65] such as intra-organizational relationships (e.g., roles among stakeholders) (*n* = 11) [11, 12, 35, 39, 42, 44, 47, 49, 59–61], inter-organizational relationships (e.g., collaboration with external stakeholders at all intervention levels) (*n* = 12) [35, 36, 39, 41, 42, 44, 50, 60–62, 64, 65], characteristics of stakeholders (e.g., description of features needed for intervention stakeholders) (*n* = 5) [36, 50, 52, 61, 64], and health systems interaction (i.e., how does the intervention interact with the health system) (*n* = 4) [49, 52, 62, 64].

### Ethics

The theme *Ethics* brings together all those sections of the frameworks that address the ethical considerations arising from the development and implementation of public health interventions. 18 of 39 (46%) frameworks included criteria and sub-criteria for the theme ethics [11, 12, 31, 33, 36, 37, 39, 41, 43–46, 52, 55–57, 64, 66]. Sub-themes were autonomy (*n* = 6) [37, 41, 43, 44, 57, 66], beneficence (*n* = 7) [11, 12, 31, 36, 41, 46, 64], harm/non-maleficence (*n* = 5) [12, 41, 52, 64, 66], and equity (*n* = 9) [12, 33, 39, 41, 43, 45, 52, 57, 64]. In addition, two frameworks described ethics without further specifications [55, 56].

### Context

The concept of *Context* arises from acknowledging the multifaceted interactions between an intervention and its context. This encompasses various layers, including legal frameworks, cultural dynamics, political landscapes, historical backgrounds, and environmental factors. 25 of the 39 (64%) included frameworks described criteria and sub-criteria related to the context [11, 12, 30, 32, 33, 35–41, 43, 47–50, 55, 57, 58, 60, 62, 64–66]. Context was divided into environmental (*n* = 11) [30, 35, 36, 39, 43, 48, 49, 55, 57, 60, 62], legal, including data protection and data security (*n* = 8) [40, 41, 47, 48, 55, 64–66], cultural (*n* = 4) [33, 35, 49, 55], political (*n* = 11) [11, 12, 33, 35–37, 43, 50, 55, 57, 58], historical (*n* = 1) [36], and general context (*n* = 2) [32, 38].

## Discussion

We identified 39 public health frameworks for the development and evaluation of interventions in primary prevention and health promotion published between 1986 and 2021. The most common aim among the included frameworks was the evaluation of interventions, followed by development, monitoring, validation, and implementation of interventions. About half of the frameworks were designed to encompass all public health interventions in primary prevention and health promotion, while the other half were more specific. The number of criteria and sub-criteria in the included frameworks varied widely, ranging from two to eleven criteria and from none to 63 sub-criteria, with no notable differences in the number of criteria and sub-criteria between generic and more specific public health frameworks observed. This variation may reflect the level of detail that can be applied when using the frameworks. The criteria and sub-criteria described in the specific public health frameworks were mostly generic and can be transferred to other contexts. For instance, the frameworks for evaluating physical activity [43] and dietary interventions [57] outlined criteria related to physical activity or nutrition only for assessing baseline and follow-up outcomes. This also applies to the framework for analysing physical activity policies [50] or economic evaluation of physical activity interventions [63], where specific criteria for physical activity were mentioned about the type of policy or program. On the other hand, the frameworks proposed by Goldsmith and colleagues [46] and van Weert and colleagues [59] described only generic criteria for evaluating a wellness or HIV prevention program.

Adapting public health programs or interventions to other populations or settings is essential to research practice translation [69]. Only a few of the included frameworks described criteria for adapting existing public health interventions (grouped under the themes *Intervention development* (intervention content and components) and *Dissemination and sustainability*, see Supplementary Material 3). One possible explanation is that adapting public health interventions to other populations or settings usually follows a stepwise process. However, these stepwise processes are mainly covered by prescriptive frameworks [23], which were excluded from this scoping review because they did not provide the information to determine the quality or integrity of health interventions.

Empirical applications of public health frameworks offer researchers and health professionals the opportunity to gain insight into how a specific framework can be applied to different health issues, populations, settings, and intervention phases (e.g., development or evaluation of an intervention). In addition, they can support researchers and health professionals in the decision-making process for choosing a suitable public health framework. One framework that is used very frequently is the RE-AIM framework [31]. In a systematic review of the application of the RE-AIM framework, 157 articles were included, the majority of which used the framework to evaluate an intervention, program, or policy [70]. However, it should be noted that empirical applications have not been published for all public health frameworks, nor can they be identified through extensive literature searches, including a review of grey literature.

Other reviews analysed more specific frameworks in public health and health care. While some categories are similar to ours, others are not: A recent review on implementation and evaluation frameworks for public health interventions specifically focused on their usability to inform co‑creation [16]. Given the focus on informing co-creation, this review concentrates on elaborating process-linked features of the frameworks, such as system thinking, partnering with stakeholders, or iterative and cyclical evaluation. A review on scaling science in health and social care identified 13 frameworks, eight specific to public health [71]. One interesting finding was that most frameworks applied a teleological lens, while only a few chose a deontological or mixed perspective. The ethical perspective is not a category we used, and it might be more challenging, as the purpose of the frameworks in our review differs from those in scaling science. A review of methodological frameworks supporting transferability and implementation identified two paradigms in the included frameworks [72]. The first paradigm is described as a linear and context-free approach to developing, transferring, and implementing interventions and solutions, implying that the exact solutions can produce the same outcomes at different sites. In contrast, the second paradigm is non-linear and systemic, assuming that effects are modified by the interaction between the solution and differing contextual factors.

### Strengths and limitations

To our knowledge, this is the first study to give an overview of existing public health frameworks for developing and evaluating interventions in primary prevention and health promotion. Further, for the scoping review, we searched five different databases. We manually searched the reference lists of the included systematic or scoping reviews to ensure that we found as many frameworks as possible.

Despite these strengths, there are a few limitations worth noting: In our scoping review of public health frameworks, there is a potential risk of overlooking valuable frameworks that might be published on non-academic websites, highlighting the need for more comprehensive and exhaustive searches in the grey literature. In addition, the search strategy (i.e., restricting search for framework, evaluation, and their synonyms only to the title, and searching only for the term framework but not models) may have resulted in not all applicable hits being identified. However, the search strategy was built to maximize sensitivity while striving for reasonable precision and was approved by a professional librarian. Additionally, we conducted a manual reference screening of relevant reviews to identify frameworks that might be relevant. Further, we explicitly didn’t include prescriptive frameworks that provide direction on the process via a series of steps or procedures (commonly named as “model”) without specific standards, principles, criteria, characteristics, or properties to determine the quality or integrity of health interventions. For instance, we did not include the Medical Research Council (MRC) framework [1] because it describes only distinct phases for developing and evaluating complex interventions. However, for our overview and summary of themes and sub-themes, we categorized them into four phases: development, implementation, evaluation, and dissemination and sustainability. Future research could focus on how criteria are described in different frameworks, addressing this gap. The categorization of sub-themes into seven themes according to the intervention phases development, implementation, evaluation, dissemination and sustainability, and none-phase-related themes (i.e., organizational aspects, context, and ethics) contains a subjective assessment based on discipline-specific knowledge and operationalization of constructs. Another limitation of our study is that the search was conducted in spring 2022. A regular update of the search may identify further frameworks for developing and evaluating public health interventions. However, our analysis already revealed a saturation of categories, so we do not anticipate a tangible benefit from doing so. Furthermore, we concentrated solely on primary prevention and health promotion frameworks, leaving room for further research into other aspects like rehabilitation, nursing, and the healthcare system within the public health domain. However, even within the primary prevention and health promotion frameworks, we found heterogeneity among studies, as some of the frameworks addressed specific health behaviors such as physical activity, which may impede the generalizability of the conclusions. Our search was limited to English, potentially overlooking relevant information in government frameworks published in other languages. This is also reflected in the origin of studies, with half of the included frameworks coming from the US. Future inquiries should consider expanding the language scope for a more inclusive understanding of global public health frameworks. Lastly, we did not calculate interrater reliability for piloting the data coding process.

### Implications for education and policymakers

The results of this scoping review have several implications for education and policymakers. Firstly, the review highlights the need for policymakers to consider the context in which interventions are developed and implemented. The frameworks identified in this review emphasize the importance of considering the environmental, legal, cultural, political, ethical, and historical context in which interventions are implemented. The frameworks also underscore the importance of considering the transfer and dissemination of intervention results and the maintenance of interventions in real-world settings. This overview of public health frameworks support policymakers, researchers, and practitioners in selecting a framework that best fits the intended purpose while ensuring that no crucial aspects of developing and evaluating public health interventions are overlooked. Depending on their available time and resources, they may choose between more or less detailed frameworks. Combining frameworks is also an option if no single framework fully meets their needs. They can also decide based on the method used to develop the framework. Researchers may validate further frameworks, especially those developed without using any methods. Moreover, given the structured compilation – organized into four phases and three non-phase-related themes – policymakers may consider combining different frameworks for a more tailored use.

## Conclusions

This scoping review provides an overview of existing public health frameworks for developing and evaluating primary prevention and health promotion interventions. From 39 included frameworks, seven overarching themes, organized into four phases (i.e., intervention development, intervention implementation, intervention evaluation, and dissemination and sustainability) and three non-phase-related themes (i.e., organizational aspects, ethics, and context), were identified to support policymakers, researchers, and practitioners in developing and evaluating public health interventions.

## Supplementary Information

Supplementary Material 1.

Supplementary Material 2.

Supplementary Material 3.

## Data Availability

The datasets used and/or analyzed during the current study are available from the corresponding author upon reasonable request.

## Abbreviations

JBI: Joanna Briggs Institute
MRC: Medical Research Council
NICE: National Institute for Health and Care Excellence
PCC: Population, Concept, Context
PRISMA-ScR: Preferred Reporting Items for Systematic Reviews and Meta-Analyses Extension for Scoping Reviews
RE-AIM: Reach, Effectiveness, Adoption, Implementation, Maintenance
WHO: World Health Organization
