# Supplementary material for: Frameworks for developing and evaluating public health interventions in primary prevention and health promotion: a scoping review
Source: BMC Public Health. 2026 Apr 11;26:1239. doi: 10.1186/s12889-026-27174-x (PMC13085540; doi:10.1186/s12889-026-27174-x)
Supplement: Supplementary file 1 — Supplementary Material 1. [file 12889_2026_27174_MOESM1_ESM.docx]

|  | Search syntax |
| --- | --- |
| **PubMed** | |
|  | ("Public Health"[Title/Abstract] OR "health promot*"[Title/Abstract] OR "prevention"[Title/Abstract]) AND (("evaluat*"[Title] OR "assess*"[Title] OR "apprais*"[Title] OR "develop*"[Title] OR "monitor*"[Title] OR "implement*"[Title] OR "evidence-based"[Title]) AND ("Framework"[Title] OR "Frameworks"[Title] OR "Guideline"[Title] OR "Guidelines"[Title] OR "Guidance"[Title] OR "Guidances"[Title])) |
|  |  |
| **Scopus** | |
|  | ( TITLE-ABS ( "Public Health"  OR  "health promot*"  OR  "prevention" ) )  AND  ( TITLE ( "evaluat*"  OR  "assess*"  OR  "apprais*"  OR  "develop*"  OR  "monitor*"  OR  "implement*"  OR  "evidence-based" ) )  AND  ( TITLE ( "Framework"  OR  "Frameworks"  OR  "Guideline"  OR  "Guidelines"  OR  "Guidance"  OR  "Guidances" ) ) |
|  |  |
| **APA PsycInfo** 1967 to April Week 1 2022 via OVID | |
| 1 | (Public Health or health promot* or prevention).ab,ti. |
| 2 | (evaluat* or assess* or apprais* or develop* or monitor* or implement* or evidence-based).ti. |
| 3 | (Framework or Frameworks or Guideline or Guidelines or Guidance or Guidances).ti. |
| 4 | 1 and 2 and 3 |
|  |  |
| **CINAHL**via EBSCOhost Research Databases | |
| 1 | TI ( "Public Health" or "health promot*" or "prevention" ) OR AB ( "Public Health" or "health promot*" or "prevention" ) |
| 2 | TI (evaluat* or assess* or apprais* or develop* or monitor* or implement* or "evidence-based") |
| 3 | TI (Framework or Frameworks or Guideline or Guidelines or Guidance or Guidances) |
| 6 | S1 AND S2 AND S3 |
|  |  |
| **IEEE** Xplore | |
|  | ((("Document Title":Health OR "Document Title":Prevention) OR ("Mesh_Terms":Health OR "Mesh_Terms":Public Health) AND ("Document Title":"evaluat*" OR "assess*" OR "apprais*" OR "develop*" OR "monitor*" OR "implement*" OR "evidence-based") AND ("Document Title":"Framework" OR "Document Title":"Document Title":"Guideline" OR "Document Title":"Document Title":"Guidance")) |
