# Supplementary material for: Frameworks for developing and evaluating public health interventions in primary prevention and health promotion: a scoping review
Source: BMC Public Health. 2026 Apr 11;26:1239. doi: 10.1186/s12889-026-27174-x (PMC13085540; doi:10.1186/s12889-026-27174-x)
Supplement: Supplementary file 3 — Supplementary Material 3. [file 12889_2026_27174_MOESM3_ESM.docx]

# Additional file 1. PRISMA-ScR checklist

| **Section** | **Item** | **PRISMA-ScR Checklist Item** | **Location and page(s)** |
| --- | --- | --- | --- |
| Title | 1 | Identify the report as a scoping review. | Title (p. 1) |
| **Abstract** |  |  |  |
| Structured summary | 2 | Provide a structured summary that includes (as applicable): background, objectives, eligibility criteria, sources of evidence, charting methods, results, and conclusions that relate to the review questions and objectives. | Abstract  (p. 2) |
| **Introduction** |  |  |  |
| Rationale | 3 | Describe the rationale for the review in the context of what is already known. Explain why the review questions/objectives lend themselves to a scoping review approach. | Background (p. 3-4) |
| Objectives | 4 | Provide an explicit statement of the questions and objectives being addressed with reference to their key elements (e.g., population or participants, concepts, and context) or other relevant key elements used to conceptualize the review questions and/or objectives. | Eligibility criteria  (p. 4) |
| **Methods** |  |  |  |
| Protocol and registration | 5 | Indicate whether a review protocol exists; state if and where it can be accessed (e.g., a Web address); and if available, provide registration information, including the registration number. | Abstract, Study design and protocol (p. 2, 4) |
| Eligibility criteria | 6 | Specify characteristics of the sources of evidence used as eligibility criteria (e.g., years considered, language, and publication status), and provide a rationale. | Eligibility criteria, Table 1  (p. 4-5) |
| Information sources | 7 | Describe all information sources in the search (e.g., databases with dates of coverage and contact with authors to identify additional sources), as well as the date the most recent search was executed. | Search strategy and databases  (p. 5) |
| Search | 8 | Present the full electronic search strategy for at least 1 database, including any limits used, such that it could be repeated. | Additional file 2 |
| Selection of sources of evidence | 9 | State the process for selecting sources of evidence (i.e., screening and eligibility) included in the scoping review. | Selection of sources of evidence, Figure 1  (p. 6-7, 23) |
| Data charting process | 10 | Describe the methods of charting data from the included sources of evidence (e.g., calibrated forms or forms that have been tested by the team before their use, and whether data charting was done independently or in duplicate) and any processes for obtaining and confirming data from investigators. | Data charting process and items (p. 7) |
| Data items | 11 | List and define all variables for which data were sought and any assumptions and simplifications made. | Data charting process and items,  Table 2  (p. 7, 25-28) |
| Critical appraisal of individual sources of evidence | 12 | If done, provide a rationale for conducting a critical appraisal of included sources of evidence; describe the methods used and how this information was used in any data synthesis (if appropriate). | N/A |
| Summary measures | 13 | *Not applicable for scoping reviews* | N/A |
| Synthesis of results | 14 | Describe the methods of handling and summarizing the data that were charted. | Data synthesis  (p. 7-8) |
| Risk of bias across studies | 15 | *Not applicable for scoping reviews* | N/A |
| Additional analyses | 16 | *Not applicable for scoping reviews* | N/A |
| **Results** |  |  |  |
| Selection of sources of evidence | 17 | Give numbers of sources of evidence screened, assessed for eligibility, and included in the review, with reasons for exclusions at each stage, ideally using a flow diagram. | Figure 1  (p. 23) |
| Characteristics of sources of evidence | 18 | For each source of evidence, present characteristics for which data were charted and provide the citations. | Table 2  (p. 25-28) |
| Critical appraisal within sources of evidence | 19 | If done, present data on critical appraisal of included sources of evidence (see item 12). | N/A |
| Results of individual sources of evidence | 20 | For each included source of evidence, present the relevant data that were charted that relate to the review questions and objectives. | Table 3, Additional file 3  (p. 29-31) |
| Synthesis of results | 21 | Summarize and/or present the charting results as they relate to the review questions and objectives. | Themes and sub-themes identified from included frameworks, Figure 2, Table 3  (p. 10-13, 24, 29-31) |
| Risk of bias across studies | 22 | *Not applicable for scoping reviews* | N/A |
| Additional analyses | 23 | *Not applicable for scoping reviews* | N/A |
| **Discussion** |  |  |  |
| Summary of evidence | 24 | Summarize the main results (including an overview of concepts, themes, and types of evidence available), link to the review questions and objectives, and consider the relevance to key groups. | Discussion (p. 13-14) |
| Limitations | 25 | Discuss the limitations of the scoping review process. | Strengths and limitations (p. 15-16) |
| Conclusions | 26 | Provide a general interpretation of the results with respect to the review questions and objectives, as well as potential implications and/or next steps. | Conclusions (p. 16) |
| Funding | 27 | Describe sources of funding for the included sources of evidence, as well as sources of funding for the scoping review. Describe the role of the funders of the scoping review. | Funding  (p. 17) |

Source: Tricco AC, Lillie E, Zarin W, O'Brien KK, Colquhoun H, Levac D, et al. Prisma Extension For Scoping Reviews (Prisma-Scr): Checklist and explanation. Ann Intern Med. 2018;169(7):467-73. doi: 10.7326/m18-0850.
